# Resilience of an Earthquake-Stricken Rural Community in Southwest China: Correlation with Disaster Risk Reduction Efforts

**DOI:** 10.3390/ijerph15030407

**Published:** 2018-02-27

**Authors:** Ke Cui, Ziqiang Han, Dongming Wang

**Affiliations:** 1 School of Public Administration, Sichuan University, Chengdu 610065, China; cuikesocialwork@163.com; 2 Institute for Disaster Management and Reconstruction, Sichuan University, Chengdu 610207, China; 3 Center for Crisis Management Research, Tsinghua University, Beijing 100084, China; 4 National Disaster Reduction Center, Ministry of Civil Affairs of China, Beijing 100124, China; wangdm@cndr.gov.cn

**Keywords:** community resilience, rural areas, China, Wenchuan earthquake

## Abstract

Disaster risk reduction (DRR) activities have given growing attention to building community resilience, but the effects of such efforts on community resilience are still under-investigated, especially in China where the concept of community resilience has only just emerged. Using the Communities Advancing Resilience Toolkit Assessment Survey, data on self-perceived community resilience were collected in 2017 from a post-disaster Chinese rural community in Yingxiu Town, which was the epicenter of the Wenchuan earthquake (Magnitude = 8.0) in the year 2008. Linear regression analyses were conducted to explore the correlations between residents’ DRR behaviors and perceived community resilience with the control of their socio-demographic characteristics including age, ethnicity, gender, education, income level, employment status and marital status. Results indicate that residents who volunteered for DRR activities, received geological disaster education, participated in evacuation drills, and reported higher income levels had a perception of higher community resilience. Practice research is suggested to help clarify the cause and effect of DRR work on the enhancement of community resilience to disasters in China and abroad. Attention is also called to the development of a Chinese indigenous community resilience concept and assessment instrument.

## 1. Introduction

Disasters—which have been defined as processes that encompass an event or a series of events [1]—often involve widespread human, material, economic, and environmental impacts. The range of negative consequences caused by disasters can potentially weaken a community’s overall well-being, even for long periods of time, and such impacts usually exceed the ability of the affected community or society to cope by only using its own resources [2]. There are four stages in the disaster management cycle: preparedness, response, recovery, and mitigation [3,4]. Traditionally, the aim of disaster management is to significantly reduce the potential losses due to hazards and ensure that the victims of disasters receive the appropriate assistance and recover from the impacts rapidly and effectively [5]. Recent consensus on disaster management highlighted the importance of pre-disaster mitigation and preparation. The United Nations Office for Disaster Risk Reduction’s (UNISDR) Hyogo Framework for Action 2005–2015 [6] has significantly shifted disaster concepts and approaches from a stance of reactive emergency response and recovery to an attitude of pro-active disaster prevention, mitigation, and preparation. This shift is also evident in the Sendai Framework for Disaster Risk Reduction 2015–2030 (Sendai Framework) adopted in March 2015 in Sendai, Japan by 187 UN members [7,8]. The goals and priority areas for action within the Sendai Framework have particularly stressed disaster risk reduction (DRR) [9]. According to the UNISDR, DRR refers to the concept and practice aim to analyze and reduce the causal factors of disasters induced by natural hazards, such as earthquake, floods, droughts, and cyclones; initiatives of DRR not only involve various disciplines including but not limited to disaster management, disaster mitigation, and disaster preparedness, but also links every part of society, government and every part of professional and private sectors [10,11]. DRR is also a significant part of sustainable development [7,12,13]. 

The 2005 World Conference on Disaster Risk Reduction not only issued the Hyogo Framework for Action 2005–2015 but also for the first time confirmed the necessity to bring the concept of “resilience” into disaster discourse [14]. Practices of were DRR thereby encouraged for building resilience to current and future disaster risks [9]. Resilience can be considered at different levels including individual, family, community, state or ecological system [1,15]. DRR programs have paid growing attention especially to community resilience across the world because communities are widely recognized as the first social units to be affected and to respond when a disaster strikes [16,17,18,19]. In the United States, for example, community resilience has become a fundamental feature of homeland security and disaster management policy and planning, and has been placed as a critical component of public health, the practice of which requires close collaboration among different sectors and groups, and their partnerships with communities [20,21,22]. 

The role of community resilience in disaster management policies and practices has received growing emphasis, but there is not yet a consensus on the definition of community resilience, which is, nonetheless, widely considered as the sustained ability of a community to withstand and recover from adversities [20,23,24]. However, community resilience is not simply a characteristic or property possessed by a community but also an ongoing dynamic process whereby resilience is enhanced by certain activities [25,26]. Community resilience has also been seen as a social process through which local communities enact collective action independently for community survival and wellbeing [27]. In a more specific manner, CARRI (Community and Regional Resilience Institute) considers community resilience as the ability to monitor threats and reduce negative effects of threats through effective response and adaption [28]. In other words, a community is a disaster resilient one when it can take mitigating and preparatory actions to resist disaster and to achieve a required level of protection [14]. Hazard mitigation, preparation, and recovery should, therefore, cultivate community disaster resilience [23]. 

At the operational level, Chandra et al. [29] identified eight essential levers of community resilience: wellness, access, education, engagement, self-sufficiency, partnership, quality, and efficiency. In this model, community resilience is composed of the social and economic well-being of a community, physical and psychological health of a population, effective risk communication, social connection and integration, and involvement of both governmental and non-governmental organizations (NGOs). Prior empirical research, such as the study of Leykin et al. [30] in Israel and the investigation of Pfefferbaum et al. [31] in an impoverished urban community in the United States revealed that the number of years living in a community, engagement with or volunteering in local entities/activities for DRR, having permanent relationships, and home ownership were positively correlated with perceived community resilience, whereas gender, home emergency preparedness, total family income, employment status, past emergency situations, and level of education had no statistically significant impact. In another study with affiliated volunteer responders in an American community, Pfefferbaum and colleagues [32] indicated that the completion of a basic training program in disaster response and the interest in deploying such a program within a community was negatively associated with the responders’ perception of community resilience. 

Disaster management has long been a major concern in China because China experiences a considerable number of natural disasters due to its vast territory, as well as complicated climate and geological conditions [33,34]. Since 2008, when the destructive Wenchuan earthquake (Magnitude = 8.0) caused approximately 87,150 casualties and enormous economic losses, a variety of natural disasters, including the Yushu earthquake (M = 7.1), Zhouqu debris flow, Lushan earthquake (M = 7.0), Maoxian landslide, and Jiuzhaigou earthquake (M = 7.0), have afflicted Chinese mainland especially in the southwest region. In the course of responding to these disasters, the disaster management capacity of China has been boosted with a noteworthy shift in disaster management policy and planning from a focus on disaster response and recovery to an equal emphasis on DRR and prevention [33,35]. A series of community-based DRR programs in disaster-prone areas have been promoted by governmental and non-governmental organizations for the enhancement of community disaster resilience [36,37,38]. These programs usually involve cross-sector risk investigations with community members, education and training, early warning and emergency response drills. The primary approaches to these activities are to increase a community’s safety awareness, preparedness for disasters, and both self-help and mutual help capacities [35].

Researchers and practitioners are still being challenged to deal with the resilience of a community at the conceptual level (i.e., defining and identifying resilient actions), the operational level (i.e., modeling the resilient behavior of communities in a framework), and the empirical level (i.e., collecting and analyzing data on community resilience) [17]. Thus, this study contributes to the understanding of community resilience in the Chinese context using recently collected empirical data. It can improve our knowledge on community resilience both theoretically and practically, as the concept of community resilience is still in its fledgling stage in China, despite the very limited studies [38,39].

This study explores the roles of current DRR activities in promoting community resilience to disasters. With a brief review of studies on community resilience and DRR activities, we reported the research methods, including the description of the study area, the instrument and measurements, the participants, and data analysis strategies. Then the descriptive analysis of the participation of DRR activities, the perception of community resilience, and the correlation between participants’ DRR behaviors and perceived community resilience were presented. Finally, the implication of this study on improving the existing resilience-building efforts for policymakers, practitioners, and academic researchers was discussed. 

## 2. Methods

### 2.1. Study Area 

The data used in this article were collected at Yingxiu town, Wenchuan, China, which was the epicenter of the 2008 Wenchuan earthquake. There are eight villages within the town, and all of them were severely affected by the 2008 earthquake with approximately 9000 of 12,000 villagers lost their lives. The survivors moved back to the town at the end of 2010 after a two-year period of recovery and reconstruction work led by the central and local governments. However, a series of secondary geological disasters, such as landslides and debris flows, had continuously afflicted the town. In particular, two major debris flows on 14 August 2010 and 10 July 2013 brought new and serious challenges to the town, prompting the Yingxiu township government to put greater efforts into disaster mitigation, prevention, and preparedness. 

A series of DRR activities have been implemented by the local government (as shown in below), collaborating with NGOs since 2013 [36]. These activities can be paired with six—i.e., wellness, access, education, engagement, self-sufficiency, and partnership—of the eight domains of community resilience proposed by Chandra et al. [29]:

(a) Building community-based (i.e., township governments, autonomous village committees, private sectors, civil society organizations, and residents) disaster response and rescue teams with clear-cut assignments of responsibilities in planning and practice (self-sufficiency and partnership);
(b) Local governments, NGOs, and residents cooperating in the compilation of community disaster risk maps and formulation of emergency response plans (engagements and partnership);
(c) Educating and training villagers about geological disasters, risk prevention techniques, and evacuation and survival skills (education);
(d) Designation of trained village volunteers to join in the local government’s work of monitoring disasters, identifying vulnerable residents, designing evacuation routes, and daily management of disaster warning systems in the community (engagement);
(e) Distribution of “information cards” and “disaster reduction cards” to inform villagers about identifying danger signals, whom to contact, and how to evacuate when an emergency occurs (education);
(f) Performing both village-based and whole town-based evacuation drills, which are usually organized by villagers both independently, and with the support and guidance of the local government and NGOs (self-sufficiency);
(g) Encouraging local NGOs to implement culturally responsive and resilience-oriented psychosocial work for women, children, and older people, i.e., vulnerable groups (wellness, access, and partnership).

### 2.2. Instrument and Measurement

#### 2.2.1. Community Resilience

Several different tools have been developed to measure community resilience by researchers [21,30,40], and every tool has its own strength and weakness. Based on sound theory and rich field tests, the Communities Advancing Resilience Toolkit (CART) Assessment Survey [22,32] is one of the most influential questionnaires. The initial version of the CART Assessment Survey contains 21 core community resilience items for addressing four interrelated domains: (1) Connection and Caring, (2) Resources, (3) Transformative Potential, and (4) Disaster Management. An expanded version was developed later with the addition of 5 items related to Information and Communication, which formed the fifth domain of the CART Assessment Survey [32]. The expanded questionnaire was translated into Chinese and validated with a sample of the Chinese population (N = 880) [41]. Given the different social, cultural, and economic contexts, as well as the uniqueness of the Chinese rural/urban communities and specific community development status in China, the Chinese version of the CART Assessment Survey was used in this study. 

The questionnaire had 26 items divided into five domains. A five-point Likert scale denoting the extent of agreement (from “1” strongly disagree to “5” strongly agree) was used for each item. The first domain reflected social bonding and connection within a community and consisted of five items with a Cronbach’s alpha test value of 0.8581 in the present study. The second domain also had five items covering the available resources within a community. The Cronbach’s alpha test value of these five items was 0.8712. Eight items were included in the third domain (Transformative Potential) with a Cronbach’s alpha test value of 0.9451. The last two domains—Disaster Management, and Information and Communication—included four items each with Cronbach’s alpha test values of 0.9123 and 0.8796, respectively. All the Cronbach’s alpha test values of the five domains were larger than 0.8, indicating good internal consistency within each domain. The mean scores within each domain were calculated to give the overall score of that domain, and the mean score of all 26 items was used as the overall community resilience score. 

#### 2.2.2. Disaster Risk Reduction Behaviors of the Participants

The Yingxiu township government has made great efforts for DRR since 2013. Through all these activities listed in Section 2.1, local people’s participation was being strongly promoted. In particular, the township government organized training sessions for villagers to help them understand geological disasters and master basic life-saving skills such as preparing house supplies for incidents. Villagers were also encouraged to join in the government’s DRR activities as volunteers. They could particularly help with identifying vulnerable residents in the community, designing evacuation routes and making community-based emergency response plans. The township government together with local NGOs performed community-based evacuation drills as well so that residents could take responsibility for disaster preparedness and be effective in reactions since they are often the first responders to an emergency. 

Four questions were designed regarding people’s participation in DRR activities to identify their DRR behaviors. The questions were: “Do you have supplies (e.g., food, drinking water, and medicine) prepared in your house to be used when a disaster occurs?”, “Have you ever volunteered in any community-based disaster prevention and mitigation activities?”, “Have you received any education on geological disasters during the past three years?”, and “Have you ever participated in an evacuation drill?” Corresponding DRR behaviors of the participants were (1) preparation of necessary materials for an emergency at home, (2) being a volunteer for DRR activities in the community, (3) receiving education on geological disasters, and (4) participating in evacuation drills. Responses to these questions were obtained by a series of check-lists in which number 1 was designated as a positive response while number 0 was designated to negative choices. All the variables were operated as dummy variables. 

#### 2.2.3. Socio-Demographic Characteristics of the Participants 

Socio-demographic characteristics, such as age, gender, ethnicity, marital status, education, employment status, and income level, were also included. Age and education were categorical variables with the level of education ranging from 1 to 5, indicating (a) illiterate, (b) primary school, (c) middle school, (d) high school, and (e) college or above. The categories for age were (a) younger than 18 years, (b) between 18 and 60 years old, and (c) older than 60 years. Gender, ethnicity, marital status, and employment status were dummy variables with one designating male, a member of a minority group, married, and employed in the agricultural sector, respectively. Since the exact amount of income might be a sensitive topic to query, the income level was obtained by self-reporting of “higher than,” “lower than,” or “almost the same as” the annual average capital disposable income in this region (10,702 RMB, Chinese Yuan).

### 2.3. Participants and Sampling 

Two hundred villagers from 200 households (one in each) in Yingxiu were randomly selected from the permanent resident population in the town. The household registration list was obtained from the township government household registration system, and 200 households were randomly selected. Then one person in each household was chosen according to their availability with a priority for the head of household. The survey was administered from July to August 2017. The villagers were initially contacted by a resident social worker, who explained the purpose of the study and asked for their participation. Of these 200 villagers, 195 agreed to participate. Then the principal researcher together with the social worker met the participants respectively at their convenience regarding time and space and gave each of the participants a hard copy of the survey questionnaire. The principal researcher and the social worker were not present while the survey questionnaire was being completed considering that the relation-oriented nature of Chinese culture might drive the participants to give positive scores to the survey [42]. Instead, the participants were given a week’s time to fill the questionnaire and simultaneously they were asked to call the social worker after finishing the survey. The social worker or the principle researcher would return to the participants and take back the questionnaire. Of the 195 distributed questionnaires, 189 (a response rate of 96.9%) were answered and returned to the researchers. Participation in this study was entirely voluntary, which means no incentives or compensation were provided to any participants. 

### 2.4. Data Analysis 

Five multivariable linear regression models were constructed to identify possible correlations between the examined DRR activities and the five CART domains. Another linear regression model was used to explore their associations with the overall community resilience score. All the analyses were conducted using the Stata software package (version 13 MP, College Station, Texas, TX, USA).

## 3. Results

### 3.1. Socio-Demographic Characteristics of the Participants 

As shown in Table 1, about 29.10% of the 189 respondents are male, 39.68% are members of minority groups, 61.90% mainly work in agriculture-related sectors, and 86.24% are married. The percentage of males is 29.10%, which is far smaller than the rate of females, possibly because in most rural villages in China, the residents are predominantly females whose husbands have migrated to the cities for better-paid jobs [43]. The minorities in this region are mainly Qiang and Zang, which constitute 21.68% and 18.00% of the respondents, respectively. As for the level of education, most participants finished middle school (50.26%), high school (25.40%) and primary school (19.58%). Only a few are college graduates (2.65%), and even fewer are illiterates (2.12%). Using the 2016 per capita disposable income (i.e., 10,702 RMB) of rural residents in this region as a standard, 45.09% of the participants reported that their income level was lower than this standard while the other 54.91% said that their income was higher. 

### 3.2. Participation in Disaster Risk Reduction Activities

The participants’ involvement in DRR activities is reported in Table 1. Among the 189 participants, 54.91% have emergency supplies prepared at home, 19.05% have volunteered for DRR activities in the community, and 45.50% have received disaster education in the past three years. Also, approximately two-thirds of the participants reported that they had participated in evacuation drills (61.90%). 

### 3.3. Community Resilience

As shown in Table 2, the perceptions of community resilience are reflected by the means and standard deviations of each of the 26 core community resilience items along with the percentage of agreement for each item, as well as the five CART domains and the overall community resilience score. The mean core community resilience item scores range from 3.26 to 3.87 (where 1 = strongly disagree, 2 = disagree, 3 = neither agree nor disagree, 4 = agree, and 5 = strongly agree). The highest mean score, as well as the highest percentage of agreement (75.66%), is associated with the survey item: “People in my community help each other”, while the lowest mean score, as well as the lowest percentage of agreement (41.27%), is associated with the statement: “My community has the resources it needs to take care of community problems”. The ranking of the mean scores of the five domains is Disaster Management (3.67 ± 0.77), Information and Communication (3.66 ± 0.74), Connection and Caring (3.54 ± 0.72), Transformative Potential (3.54 ± 0.73), and Resources (3.45 ± 0.76). The average of the overall community resilience score is 3.58 (±0.68).

### 3.4. The Correlation of Disaster Risk Reduction Activities and Community Resilience

The OLS (ordinal least squares) regression results of the correlation between the DRR activities and community resilience scores with control of the basic socio-demographic variables are reported in Table 3. Specifically, participation in emergency evacuation drills and geological disaster education is positively associated with all the five domains. Participation in emergency response drills has a relatively stronger correlation. Moreover, volunteering for community-based DRR activities is positively correlated with all the community resilience domains, but only the correlations with the domains of Connection and Caring, Transformative Potential, and Information and Communication are statistically significant. Preparation of emergency supplies at home is significantly and positively associated with the Resources domain of community resilience but not with other domains. Meanwhile, it is worth noting that income level is also positively associated with all the community resilience domains except the domain of Connection and Caring, and their correlations are significant. Regarding the overall community resilience, significant correlations are found for income level and the experience of being a volunteer in DRR activities in the community, as well as participation in geological disaster education and evacuation drills. Additionally, within all the statistically significant predictors of the overall community resilience, participation in evacuation drills has the most robust correlation while the correlation of income level was the lowest.

## 4. Discussion

This article presents a study of the community resilience of a typical post-disaster and also disaster-prone community in rural China. Based on the data collected using the CART Assessment Survey, several linear regression models were performed to explore the correlations between the various community resilience scores (i.e., scores of five CART domains and the overall community resilience score as perceived by those who participated in the survey) and individual characteristics (including socio-demographics and DRR behaviors). The fitness of these models were appropriate with adjusted *R*^2^ values ranging from 0.297 to 0.188 [44].

The scores for core resilience items, CART domains, and the overall community resilience are all above 3 (i.e., neither agree nor disagree) but below 4 (i.e., agree). This is roughly similar to the assessment results of a prior study based on a representative sample from ten rural communities in Sichuan Province [39]. What is also consistent with this study is related to the primary community resilience strength and weakness. Primary community resilience strength was identified as the survey item with the highest percentage of the agreement; while the community resilience weakness was identified as the survey item with the lowest percentage of agreement [31]. Therefore, the primary community resilience strength of this study is associated with survey item 4 “People in my community help each other”, and the primary community resilience weakness is associated with survey item 6 “My community has the resources it needs to take care of community problems”.

Previous research has already stressed the scarcity of community resources, such as inadequate infrastructure, underdeveloped transportation, poor communication systems, and the absence of partnerships with private sectors or civil society organizations, for disaster prevention and mitigation in rural China as compared with urban neighborhoods [19]. However, the community under study is not without advantages, such as close connection and mutual care, as reflected by the primary community resilience strength. One explanation for the observed strength could be the “geography-based acquaintances society” characterized by close connection and mutual help among community members, especially in rural China [45]. The study of Zheng et al. [39] reinforced this idea as rural villagers scored higher than urban residents for items in the Connection and Caring domain (*p* < 0.05). The relations of mutual support among neighbors is a primary component of social capital, the role of which in the enhancement of community resilience has already been widely recognized and promoted [7,32]. Therefore, the continuous investment of resources in rural areas with effective use of local strengths in a culturally responsive manner is suggested for DRR policy, planning, and practice.

Unlike prior studies in China focusing on preparedness activities [46,47], this paper highlighted the importance of DRR activities in promoting community resilience. In particular, those voluntarily involved in community-based DRR activities are more likely to have a more positive perception of their community’s disaster resilience. This finding is in line with the study of Pfefferbaum et al. [31], who found a positive correlation between engagement with local DRR activities and perceived community resilience in an impoverished urban community in the United States. One explanation may be that such engagement simultaneously increases residents’ awareness of their community’s situation and promoting the realization of their capacities to make a difference to the community. In turn, participants’ awareness of their self-efficacy and knowledge of disaster preparedness (i.e., social cognitive factors) might potentially encourage their future engagement in the resilience-building campaign [48]. Shaw [49] also argued for the importance of community participation in DRR for both the process and content as the main actors, since the community will ultimately benefit from improved disaster resilience and development. 

This study also reveals that people who received disaster education would have a perception of higher community resilience than those who did not receive such education, but this finding is not supported by a study with a group of affiliated volunteer responders in an American community [32]. The latter study found the completion of basic training in disaster response was negatively associated with participants’ perceptions of communities’ Transformative Potential. Disaster education/training programs that differ in their contents or processes may be the cause of such a discrepancy. In any case, the design of community-specific DRR educational programs has already been recognized as a necessary component of DRR planning [50]. Community-based evacuation drills is another intrinsic component of DRR programs worldwide [18,35]. Particular attention should be given to the comparatively high percentage of people who had ever participated in evacuation drills (62%), because it may reflect some extent a satisfactory degree of community engagement at Yingxiu town. Moreover, among all the four DRR activities in this study, the percentage of people who had participated in evacuation drills was the highest. A study of the Great Shake Out campaign in Southern California also found that the majority of respondents (71%) had participated in the drop, cover and hold drill while they seemed much less interested in other activities such as joining MySpace (0%) and Facebook (3%) [48]. The positive correlations between the participation of people in evacuation drills and their perceived community resilience as uncovered by this study, may have reinforced current DRR planning and supported the further development of relevant programs [18]. 

In the same vein, the preparation of emergency supplies such as food and medicine at home is positively related to the perception of community resilience among this study’s participants, but the correlation is only significant in the domain of Resources. This differs from the findings of a previous study conducted in Israel [30], which indicated no statistically significant correlation between home emergency preparedness and community resilience. The reasons for this contrary finding may be due to the differences between the Chinese and Israeli cultures, and in the measurements of community resilience that were used. The CART Assessment Survey was adopted in this study while the Conjoint Community Resiliency Assessment Measure was used in Israel’s study. Moreover, of the four DRR activities in the current study, preparing emergency supplies at home involves less community interaction than the other three and has the least correlation with the five domains of the CART. It is also the only one of the four DRR activities that reveal no significant correlation with the overall community resilience. This result may imply that the underlying factor promoting the individual perception of community resilience to disaster can be active community interaction, especially given that prior research in other post-disaster rural communities has already found a significant positive correlation between placed-based social cohesion and perceived community resilience [51]. In addition, provided that community relations or networks are primary components of social capital, future research could examine the association between social capital and perceived community resilience; it is also worth going one step further to explore if anything that increases social capital within community could have the same effect in promoting community resilience.

Higher income levels reported by the participants also significantly contributed to their perception of community resilience. This might be partly because participants with higher income were more likely to participant in resilience-building activities [52]. The economic well-being of a community has already been widely recognized as a core component of community resilience [17,53,54]. Zhu [55] argued that the extent to which a community could endure the impact of or recover from a disaster largely relied on its economic condition, such as economic capital, income security, and multiple income structures. At the family level, evidence suggests that families with better economic status before a disaster would be more likely to bounce back [56]. Therefore, investments in boosting the capacity of the individual household by providing financial assistance and technical support seemed necessary for better recovery after a disaster [57]. Nonetheless, while lower income may cause families in a shortage of financial assets, it does not necessarily reduce their relational and social support assets [58]. This may explain why participants from economically disadvantaged communities will perceive a lack of resources but a strong sense of attachment at the same time [31]. In fact, sense of community and traditions of mutual help have already been seen as key assets in many local neighborhoods especially where people feel they are in need of protection to avoid economic instability [59]. For people with lower incomes, therefore, the community as a major source of social support and mutual help may be a more important asset compared to people with higher incomes. However, this study failed to support the correlations between age, ethnicity, gender, education, employment status, and marital status with an individual’s perception of community resilience. These results are consistent with previous research findings [30,31,32].

Besides the contributions discussed above, this study did have at least three limitations. First, although the findings from this cross-sectional survey would be instructive for an understanding of community resilience in rural China, what the study presented was the statistical correlations between research participants’ DRR activities, socio-demographic factors, and their perceived community resilience. Thus, practice research of quasi-experimental research design or randomized controlled trials into DRR programs would help clarify the causal relationship between specific DRR activities and community disaster resilience. Second, we used a relatively small sample from one of the most disrupted towns in the 2008 Wenchuan earthquake. Studies covering larger population in rural and urban communities in China are needed to provide more representative knowledge on community resilience. Last but not least, we only used one tool (CART) in this study to assess people’s perception of community resilience. Investigations using other alternative community resilience assessment tools, such as Chang’s framework focusing on physical aspect [60,61], or Cutter’s indicators [53,62], to cross-check the reliability and validity of varied tools in assessing community resilience will provide more knowledge about resilience from the theoretical perspective.

## 5. Conclusions

This study has examined the current status of community resilience in Yingxiu Town, an earthquake-stricken rural community in Southwest China, and explored the correlations between residents’ DRR behaviors and perception of their community’s disaster resilience. The results indicate that residents who volunteered in DRR activities and received geological disaster education, as well as those who participated in disaster evacuation drills, were more likely to have a perception of higher overall community resilience. Another factor that would contribute to this perception was income level. Future research is recommended to help clarify the cause and effect of the prevalent DRR work in enhancing community disaster resilience in China and abroad. The development of a Chinese indigenous community resilience concept and assessment instrument is also recommended.

## Figures and Tables

**Table 1:** Descriptive analysis of disaster risk reduction activities and socio-demographic characteristics (N = 189).

Variables	Frequency	Percentage
Having emergency supplies	104	54.91
Being a volunteer	36	19.05
Attended disaster education	86	45.50
Having participated in evacuation drills	117	61.90
Being male	55	29.10
Be minority	75	39.68
Income above the average	104	54.91
Main work as agricultural-related	117	61.90
Married	163	86.24
Age (years)		
<18	6	3.17
18–60	166	87.83
>60	17	8.99
Education		
Illiterate	4	2.12
Primary	37	19.58
Middle	95	50.26
High	48	25.40
College+	5	2.65

**Table 2:** Core community resilience items by domains and perceptions of community resilience.

Domain/Item/Overall Community Resilience	Mean (SD)	Percentage of Agreement (%)
CART (Communities Advancing Resilience Toolkit) Domain 1: Connection and Caring	3.54 (0.72)	-
1. People in my community feel like they belong to the community.	3.49 (0.90)	49.21
2. People in my community are committed to the well-being of the community.	3.42 (0.83)	49.74
3. People in my community have hope about the future.	3.46 (0.98)	54.50
4. People in my community help each other.	3.87 (0.80)	75.66
5. My community treats people fairly no matter what their background is.	3.47 (0.95)	53.97
CART Domain 2: Resources	3.45 (0.76)	-
6. My community has the resources it needs to take care of community problems.	3.26 (0.97)	41.27
7. My community has effective leaders.	3.40 (1.02)	49.21
8. People in my community are able to get the services they need.	3.42 (0.89)	49.74
9. People in my community know where to go to get things done.	3.48 (0.89)	55.03
10. My community supports programs for children and families.	3.72 (0.88)	66.14
CART Domain 3: Transformative Potential	3.54 (0.73)	-
11. My community works with organizations and agencies outside the community to get things done.	3.57 (0.78)	56.08
12. People in my community communicate with leaders who can help improve the community.	3.57 (0.85)	58.73
13. People in my community are aware of community issues that they might address together.	3.58 (0.84)	58.73
14. People in my community discuss issues so they can improve the community.	3.54 (0.90)	55.56
15. People in my community work together on solutions so that the community can improve.	3.70 (0.85)	64.55
16. My community looks at its successes and failures so it can learn from the past.	3.58 (0.84)	59.26
17. My community develops skills and finds resources to solve its problems and reach its goals.	3.41 (0.90)	50.26
18. My community has priorities and sets goals for the future.	3.39 (0.94)	49.74
CART Domain 4: Disaster Management	3.67 (0.77)	-
19. My community tries to prevent disasters.	3.66 (0.87)	65.61
20. My community actively prepares for future disasters.	3.62 (0.89)	61.90
21. My community can provide emergency services during a disaster.	3.71 (0.82)	66.67
22. My community has services and programs to help people after a disaster.	3.70 (0.86)	66.14
CART Domain 5: Information and Communication	3.66 (0.74)	-
23. My community keeps people informed (via television, radio, newspaper, Internet, phone, neighbors) about issues that are relevant to them.	3.68 (0.91)	64.55
24. If a disaster occurs, my community provides information about what to do.	3.68 (0.84)	66.67
25. I get information/communication from my community to help with my home and work life.	3.57 (0.88)	60.85
26. People in my community trust public officials.	3.70 (0.81)	64.02
Overall Community Resilience	3.58 (0.68)	-

**Table 3:** Linear regression results for community resilience.

	Community Resilience Domains					Overall
Variables	Connection and Caring	Resources	Transformative Potential	Disaster Management	Information and Communication	
Emergency supplies	0.088 (0.104)	0.233 * (0.104)	0.117 (0.100)	0.184 (0.107)	0.139 (0.104)	0.147 (0.091)
Being a volunteer	0.286 * (0.126)	0.234 (0.126)	0.313 * (0.122)	0.250 (0.130)	0.261 * (0.126)	0.275 * (0.111)
Disaster education	0.337 ** (0.110)	0.285 * (0.110)	0.242 * (0.107)	0.231 * (0.114)	0.307 ** (0.110)	0.277 ** (0.097)
Evacuation drills	0.305 * (0.119)	0.376 ** (0.119)	0.420 *** (0.115)	0.394 ** (0.123)	0.364 ** (0.119)	0.377 *** (0.104)
Age	0.093 (0.147)	−0.086 (0.147)	−0.128 (0.142)	−0.142 (0.152)	−0.094 (0.147)	−0.074 (0.129)
Ethnicity	0.029 (0.102)	−0.038 (0.102)	0.144 (0.098)	0.063 (0.105)	0.036 (0.102)	0.058 (0.089)
Gender	−0.008 (0.108)	−0.078 (0.108)	−0.038 (0.104)	−0.125 (0.111)	−0.038 (0.108)	−0.053 (0.095)
Education	−0.068 (0.065)	−0.066 (0.065)	−0.032 (0.063)	−0.107 (0.067)	−0.096 (0.065)	−0.067 (0.057)
Income level	0.176 (0.098)	0.294 ** (0.098)	0.258 ** (0.095)	0.295 ** (0.101)	0.255 * (0.098)	0.254 ** (0.086)
Employment status	−0.037 (0.104)	−0.073 (0.104)	−0.016 (0.101)	−0.121 (0.108)	−0.004 (0.104)	−0.045 (0.092)
Marital status	−0.035 (0.142)	−0.119 (0.142)	0.095 (0.137)	0.020 (0.147)	0.147 (0.142)	0.025 (0.125)
adj. *R*^2^	0.188	0.275	0.274	0.247	0.241	0.297

Standard errors in parentheses. * *p* < 0.05, ** *p* < 0.01, *** *p* < 0.001.
